# KCNJ2/HIF1α positive-feedback loop promotes the metastasis of osteosarcoma

**DOI:** 10.1186/s12964-023-01064-w

**Published:** 2023-03-02

**Authors:** Mao Shen, Runsang Pan, Shan Lei, Lu Zhang, Changhua Zhou, Zhirui Zeng, Yingjie Nie, Xiaobin Tian

**Affiliations:** 1 Department of Orthopedics, The Affiliated Hospital of Guizhou Medical University, Guiyang, 550009 Guizhou China; 2 School of Basic Medicine, Guizhou Medical University, Guiyang, 550009 Guizhou China; 3 School of Clinical Medicine, Guizhou Medical University, Guiyang, 550009 Guizhou China; 4 The Central Laboratory, Guizhou Provincial Peoples Hospital, Guiyang, 550009 Guizhou China

**Keywords:** HIF1α, KCNJ2, Metastasis, Osteosarcoma

## Abstract

**Background:**

Early metastasis is a hallmark of osteosarcoma (OS), a highly common type of malignant tumor. Members of the potassium inwardly rectifying channel family exert oncogenic effects in various cancers. However, the role of the potassium inwardly rectifying channel subfamily J member 2 (KCNJ2) in OS is unclear.

**Methods:**

The expression of KCNJ2 in OS tissues and cell lines was measured using bioinformatic analysis, immunohistochemistry, and western blotting. Wound-healing assays, Transwell assays, and lung metastasis models were used to analyze the effects of KCNJ2 on mobility of OS cells. The molecular mechanisms linking KCNJ2 and HIF1α in OS were explored by mass spectrometry analysis, immunoprecipitation, ubiquitination detection, and chromatin-immunoprecipitation quantitative real-time polymerase chain reaction.

**Results:**

KCNJ2 was found to be overexpressed in advanced-stage OS tissues, as well as in cells with high metastatic potential. High expression of KCNJ2 was associated with a shorter survival rate of OS patients. KCNJ2-inhibition repressed the metastasis of OS cells, whereas KCNJ2-elevation induced the opposite effects. Mechanistically, KCNJ2 binds to HIF1α and inhibits its ubiquitination, thus increasing the expression of HIF1α. Interestingly, HIF1α binds directly to the KCNJ2 promoter and increases its transcription under hypoxic conditions.

**Conclusion:**

Taken together, our results indicated that a KCNJ2/HIF1α positive feedback loop exists in OS tissues, which significantly promotes OS cell metastasis. This evidence may contribute to the diagnosis and treatment of OS.

Video Abstract

**Supplementary Information:**

The online version contains supplementary material available at 10.1186/s12964-023-01064-w.

## Introduction

Osteosarcoma (OS) is an extremely malignant bone tumor that develops from mesenchymal cells, with high mortality in children and adolescents [1]. It typically metastasizes early. OS is diagnosed with macroscopic evidence of metastasis in 15–20% of cases, most commonly (85–90%) in the lungs [2, 3]. Previous studies have indicated that patients without metastases who were treated with local surgery and neoadjuvant chemotherapy had a survival rate of up to 70%, whereas those with metastasis had a survival rate of < 30% [4]. Therefore, exploration of key mediators involved in metastasis may contribute to OS diagnosis and therapy.

In solid tumors, hypoxia commonly triggers the activation of various signaling pathways, causing cancer cells metastasis [5]. HIF-1α is a transcription factor that directly reacts to hypoxia and helps cells adjust to their environment [6, 7]. Under normoxic conditions, most HIF-1α proteins would be degraded by the von Hippel–Lindau tumor suppressor [8], while a few HIF-1α proteins would escape from degradation with the help of a series of protective proteins, such as ubiquitin-specific peptidase 22 [9] and BCL2-associated transcription factor 1 [10]. Previous studies have indicated that HIF-1α was up-regulated in OS tissues, and was associated with poor prognosis [11, 12]. HIF-1α contributes to the metastasis of OS by promoting angiopoiesis, up-regulating metalloprotease levels, and enhancing the epithelial–mesenchymal transition phenotype [13, 14]. Therefore, exploration of molecular mechanisms in the HIF-1α regulation network would contribute to dig biomarkers for OS.

Inwardly rectifying potassium channel subfamily J member 2 (KCNJ2) is a member of the inwardly rectifying potassium channel family [15]. Under physiological conditions, these channels are located in the cytomembrane and regulate the potassium ion balance in cells by promoting potassium influx [16]. However, inwardly rectifying potassium channels mostly translocate to the cytosol and nucleus in cancer cells and regulate biological processes via different molecular mechanisms. The oncogenic effects of inwardly rectifying potassium channel family members have been demonstrated in various cancers. For example, KCNJ12 can bind to RELA protein in the nucleus, activate the NF-kB signaling pathway, and promote prostate cancer cell proliferation [17]. Breast cancer cells show high levels of cytoplasmic KCNJ3 expression, which is positively associated with lymph node invasion [18]. KCNJ2 is upregulated in small-cell lung cancer cells, and enhance multidrug resistance by activating the RAS/MAPK pathway [19]. However, to date, the role of KCNJ2 in OS has been poorly studied.

We explored the clinical value, biological functions, and molecular mechanisms of KCNJ2 in OS. We demonstrated that a KCNJ2/ HIF1α positive-feedback loop exists in OS tissues, which significantly promotes the metastasis of OS cells. Our results suggest that the KCNJ2 protein may serve as a novel biomarker and therapeutic target for OS.

## Materials and methods

### Clinical sample collection and tissue ethics

The 64 OS tissue samples utilized in the present study were obtained from Affiliated Hospital of Guizhou Medical University (Guiyang, Guizhou, China), with the approval by the Human Ethics Committee of Guizhou Medical University (approval number: 2021093). Written informed consent was obtained from all donor patients. Chemotherapy and radiotherapy were not administered prior to tissue collection. Among the 64 OS patients, 42 had been diagnosed with stage I–II (Enneking), while 24 had been diagnosed with stage III (Enneking). All tissues were stored at -80℃ until used for experiments.

### Identification of key genes associated with OS cell metastasis

Two gene expression matrices, GSE18947 and GSE49003, of OS cells with high and low metastatic ability, were accessed from Gene Expression Omnibus (GEO; https://www.ncbi.nlm.nih.gov/gds). The edgeR package [23] was used to analyze the differentially expressed genes (DEGs) between OS cells with high and low metastatic ability. |logFC|≥ 1 and adjusted *P* value < 0.05 were set as the threshold for significant differential expression. Overlapping DEGs, identified in both profiles, were used for further analysis.

### Immunohistochemistry method

Immunohistochemistry (IHC) was used to evaluate KCNJ2 and HIF1α expression in OS tissues. Briefly, OS tissue sections were successively deparaffinized in dimethylbenzene and rehydrated in an ethanol gradient. Following antigen removal with citrate buffer (pH 6.0), the sections were incubated with 3% H_2_O_2_ and then 5% BSA. The anti-KCNJ2 (1:50; Cat No: 19965-1-AP, Proteintech, Wuhan, China) and anti-HIF1α (1:100; Cat No: 20960-1-AP, Proteintech) primary antibodies were added to sections at 4 ℃ for 16 h. Sections were washed twice with phosphate-buffered saline (PBS), incubated for 1 h with secondary antibodies, and then stained with DAB. IHC results were calculated by multiplying the degree of intensity by positive rates. In terms of staining intensity, the scores were assigned as 0–3 (no staining, weak staining, moderate staining, and high staining), while positive rates were scored as 0–4 (no staining, 10–50%, 50–80%, and > 80%).

### Quantitative real-time polymerase chain reaction

Total RNA in OS tissues was isolated using TRIZOL reagent (Yeasen, Shanghai, China), and the concentration of total mRNA determined spectrophotometrically. A PrimeScript™ RT Reagent Kit (Thermo Fisher Scientific, Waltham, MA) was used to synthesize 800 ng of cDNA extracted from each sample. Finally, polymerase chain reaction (PCR) was performed to detect the mRNA levels of *KCNJ2* and *HIF1A* in OS tissues and cells using SYBR Green Abstart One Step RT-PCR Mix (Sangon Biotech, Wuhan, China). β-actin was used as a loading control. The primers used for quantitative real-time PCR (qRT-PCR) amplification of *KCNJ2* and *HIF1A* were as follows:

*KCNJ2*-forward: 5'-GTGCGAACCAACCGCTACA-3';

*KCNJ2*-reverse: 5'-CCAGCGAATGTCCACACAC-3';

*HIF1A-*forward: 5'-GAACGTCGAAAAGAAAAGTCTCG-3';

*HIF1A*-reverse: 5'-CCTTATCAAGATGCGAACTCACA-3';

*ACTB*-forward: 5'-CATGTACGTTGCTATCCAGGC-3';

*ACTB*-reverse: 5'-CTCCTTAATGTCACGCACGAT-3'.

### Western blotting

High-intensity RIPA lysis buffer (Nanjing Jiancheng Bioengineering Institute, Nanjing, China) containing 1/100 phenylmethanesulfonyl fluoride (Nanjing Jiancheng Bioengineering Institute) was used to extract total protein from each sample. A bicinchoninic acid detection kit (Nanjing Jiancheng Bioengineering Institute) was used to measure the protein concentrations. Sodium dodecyl sulfate–polyacrylamide gel electrophoresis (Meilunbio, Dalian, China) was used to separate protein samples. The separated protein samples in the gel were then transferred to a polyvinylidene fluoride membrane (Millipore, St Louis, MO). Membranes were blocked with 5% skim milk powder (Solarbio, Guangzhou, China) and incubated with primary antibodies, including those against KCNJ2 (1:500; Cat No: 19965-1-AP, Proteintech), HIF1α (1:1000; Cat No: 20960-1-AP, Proteintech), CA9 (1:1000; Cat No: 11071-1-AP, Proteintech), HA (1:1000; Cat No: 66006-2-Ig, Proteintech), and β-actin (1:10,000; Cat No: AC026, ABclonal, Wuhan, China) for 12 h at 4 ℃. After washing the membrane twice with TBST, it was incubated with horseradish peroxidase-conjugated secondary antibodies, followed by chemiluminescence reagents to detect protein bands (Boster, Wuhan, China). β-Actin expression was used as a loading control.

### Cell culture and transfection

Human normal osteoblasts (hFOB1.19), normal bone marrow stromal cells (BMSC), and OS cells (MG63, Saos2, U2OS, HOS, KHOS, MNNG/HOS, SJSA-1, and 143B) were purchased from the American Type Culture Collection (Manassas, VA). All cells were cultured in Dulbecco’s modified Eagle’s medium (DMEM; Hyclone, Logan, UT) with 10% fetal bovine serum (FBS, Hyclone) at 37℃, under 5% CO_2_. The *KCNJ2*-overexpression lentivirus was constructed by subcloning the PCR-amplified full-length human *KCNJ2* cDNA into the pMSCV retrovirus vector, whereas human *KCNJ2*-targeting short hairpin RNA (shRNA) oligonucleotide sequences were cloned into the pSuper-retro-puro vector to generate pSuper-retro-KCNJ2-RNAis. The negative control (NC) sequences and two targeting *KCNJ2*-shRNA sequences were as follows: NC, 5ʹ-CACCGTTCTCCGAACGTGTCACGTCAAGAGATTACGTGACACGTTCGGAGAATTTTTTG-3ʹ; sh1-KCNJ2, 5ʹ-CACCGGTGGATGCTGGTTATCTTCTTTCAAGAGAAGAAGAT AACCAGCATCCACCTTTTTTG-3ʹ; sh2-KCNJ2, 5ʹ-CACCGCTCCTCAAATCCAGAATTACTTCAAGAGAGTAATTCTGGATTTGAGGAGCTTTTTTG-3ʹ. The targeting *HIF1A*-siRNA (si-HIF1α) and the corresponding NC were obtained from iGenebio (Beijing, China): NC, 5ʹ-UUCUCCGAACGUGUCACGU-3ʹ; and si-HIF1α, 5ʹ-CAAGUAGCCUCUUUCACAA-3ʹ. Lentivirus and siRNA transfections were conducted using polybrene (Thermo Fisher Scientific) and lipo2000 (Thermo Fisher Scientific), respectively. To obtain stable overexpression/knockdown cell lines, cells were selected for 2 weeks with 1.0 μg/ml puromycin after 72 h of transfection. The parameters set as normoxic condition was 21% O_2_, 74% N_2_ and 5% CO_2_, and the parameters set as hypoxic condition was 1% O_2_, 94% N_2_ and 5% CO_2_ [20–22]_._

### Wound healing assay

OS cells were cultured in six-well plates at 37 °C to 95% confluence. Prior to construction of wound, cells were pretreated with 10 µg/ml mitomycin C for 30 min. Cell monolayer wounds were constructed using a 200-μL pipette tip. Then, following washing twice with PBS to eliminate floating cells, fresh FBS-free DMEM medium was added. A microscope with an inverted optical system was used to monitor wound status at 0 and 48 h (Olympus, Shinjuku, Japan).

### Transwell assay

Transwell cell culture inserts (BD Biosciences, Franklin Lakes, NJ), pre-coated with 8% Matrigel (BD Biosciences), were used to measure cell invasion. In brief, 200 μL cell suspension containing 1 × 10^4^ OS cells (pretreated with 10 µg/ml mitomycin C for 30 min) was placed into the upper chamber, and 700 μL DMEM medium containing 10% FBS in the lower chamber. Then, the Transwell plates were cultured in 37 ℃ for 48 h. Thereafter, the upper chambers were removed and immersed in 4% paraformaldehyde (Boster) for 20 min to fix the cells. Then, cells were stained with 1% crystal violet solution for 15 min. After washing twice with PBS and scrubbing away noninvasive cells, the chamber conditions were recorded using an optical inverted microscope (Olympus; magnification times 100 ×).

### Lung metastasis model

This study was approved by the Ethics Committee of Guizhou Medical University for Animal Experiments (approval number: 2100602). A total of 1 × 10^6^ 143B cells in 100 µL PBS were injected into the caudal vein of nude mice (*n* = 5 per group). Mice were sacrificed after 10 weeks and the lung tissues were dissected. The metastatic foci per lung were counted after hematoxylin and eosin staining.

### Immunoprecipitation

Total protein was extracted from in 143B cells using the mild RIPA lysis buffer (Nanjing Jiancheng Bioengineering Institute) containing 1/100 phenylmethanesulfonyl fluoride. Anti-KCNJ2 antibodies (1:50; Cat No. ab85492, Abcam, Cambridge, MA) were incubated with the protein lysate to generate antigen–antibody complexes. A + G magnetic beads (Yeasen, Shanghai, China) were used to immunoprecipitate the antigen–antibody complexes. The precipitate was analyzed by liquid chromatography/mass spectrometry and western blotting.

### Protein degradation experiments

The 143B cells were treated with 1 μM cyclohexane (CHX; MCE, Wuhan, China) to inhibit the transcription of proteins for 0, 1.5, 3.0, 4.5 and 6.0 h. Then, protein in cells were collected and used to perform western blotting. The degradation rates of HIF1α in 1.5, 3.0, 4.5 and 6.0 h was calculated through normalizing to the expression of KCNJ2 in 0 h.

### Ubiquitination experiments

An HA-labeled ubiquitin molecule was constructed by Genechem Bio (Shanghai, China) and transfected into 143B cells, along with an empty vector or an KCNJ2-overexpression vector. After 48 h of transfection, 1 μM CHX and 10 μM MG132 were added and the cells incubated for another 4 h. The cells were then lysed and anti-HIF1α antibodies (1:50) were used to precipitate HIF1α. The ubiquitination level of HIF1α was detected using anti-HA antibodies.

### Chromatin coprecipitation

A chromatin coprecipitation (CHIP) assay was performed using an immunoprecipitation kit (Thermo Fisher Scientific) according to the manufacturer's protocol. Cells were crosslinked with formaldehyde, and then DNA sequences (200–500 bp) were produced by sonication. Immunoprecipitation was performed using anti-HIF1α or anti-IgG antibodies. The precipitated DNA of the putative HIF1α-bound *KCNJ2* promoter was amplified using qRT-PCR.

### Dual luciferase reporter experiments

Full-length wildtype (WT) and hypoxia-response element (HRE)-mutated *KCNJ2* promoter sequences were cloned into the luciferase reporter plasmid pGL3-basic (Genechem, Shanghai). Luciferase reporter plasmids were transfected into 143B cells with *HIF1A*-knockdown and NC cells. After 24 h of transfection, the cells were cultured under normoxic and hypoxic (1% O_2_) conditions. Luciferase activity was measured using the Dual-Luciferase Reporter Assay System (Promega, Madison, WI).

### Statistical analysis

Prism v 6.0 (GraphPad Inc., La Jolla, CA) was used to conduct statistical analysis. The difference between the high-KCNJ2 and low-KCNJ2 groups was analyzed by Kaplan–Meier survival analysis, and *P* < 0.05 was set as the significant cut-off. The hazard ratio (HR) was detected by Mantel–Haenszel analysis via fitting and comparing the death rate in the patients of high-KCNJ2 and low-KCNJ2 groups. The relationship between KCNJ2 and HIF1α was analyzed by Pearson correlation analysis, and significance was defined as *r* > 0.3 and *P* < 0.05. The differences between the two groups were analyzed using the unpaired *t*-test, while those in the multiple groups were analyzed using one-way analysis of variance. Statistical significance was set at *P* < 0.05.

## Results

### KCNJ2 expression is associated with metastasis in OS

**Fig. 1:**
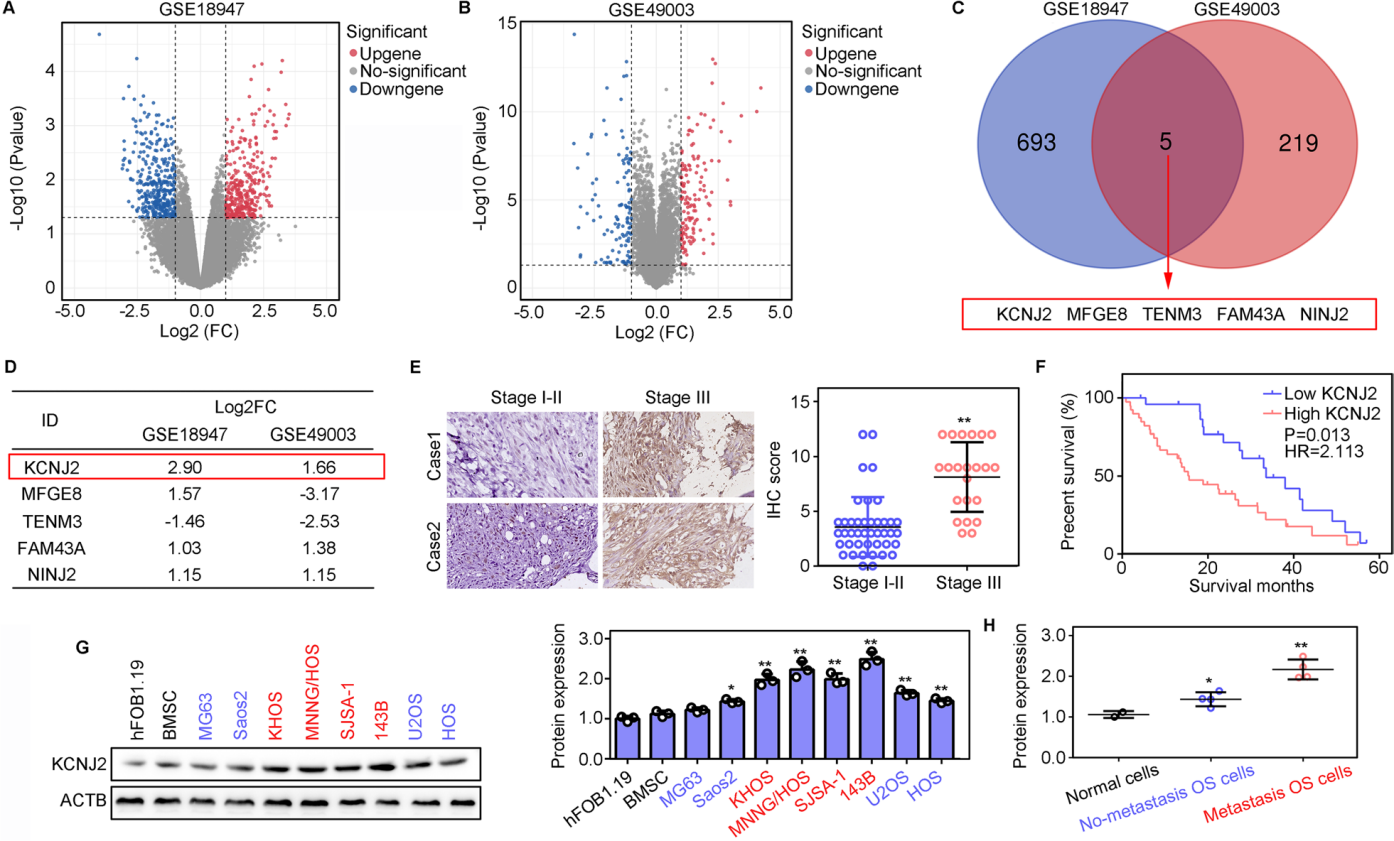
KCNJ2 expression is associated with metastasis in osteosarcoma (OS). **A** Differentially expressed genes (DEGs) between OS cells with high and low metastatic ability in the GSE18947 cohort. **B** Differentially expressed genes (DEGs) between OS cells with high metastatic ability and low metastatic ability in the GSE49003 cohort. **C** Overlapped genes in two cohorts. **D** Change-fold and *P* value of overlapped DEGs. **E** Expression of KCNJ2 in OS tissues provided by patients diagnosed with stage I–II or stage III OS. **F** Kaplan–Meier survival analysis for OS patients with high and low *KCNJ2* expression. **G**, **H** The protein levels of KCNJ2 in normal cells (hFOB1.19 and BMSC), OS cells with strong metastatic ability (KHOS, MNNG/HOS, SJSA-1, and 143B) and OS cells with low metastatic ability (MG63, Saos2, U2OS, and HOS). *, *P* < 0.05; **, *P* < 0.01

To explore key genes involved in the metastasis of OS cells, we analyzed the gene expression differences between OS cells with high metastatic capacity and those with low metastatic capacity, based on two gene expression profiles, GSE18947 and GSE49003, from the GEO database. A total of 698 and 224 DEGs were found in the GSE18947 (Fig. 1A) and GSE49003 (Fig. 1B) profiles, respectively. After intersection analysis, we found that five DEGs, including *KCNJ2, MFGE8, TENM3, FAM43A,* and *NINJ2*, overlapped in these two profiles (Fig. 1C). Among them, *KCNJ2* showed the most significant and consistent changes in both profiles (Fig. 1D). Therefore, we focused on *KCNJ2.* We found that the KCNJ2 protein expression was elevated in the advanced stage (III) as compared to that in the early stage (I–II) OS (Fig. 1E). Moreover, we found that OS patients with high KCNJ2 expression had shorter survival rates (hazard ratio: 2.113; Fig. 1F). Finally, we performed western blotting to measure the KCNJ2 protein levels in normal cells (hFOB1.19 and BMSC), in OS cells with strong metastatic ability (KHOS, MNNG/HOS, SJSA-1, and 143 B), and OS cells with a low metastatic ability (MG63, Saos2, U2OS, and HOS). The KCNJ2 protein expression level was highest in OS cells with a strong metastatic ability (Fig. 1G, H). This evidence suggests that KCNJ2 is involved in OS cell metastasis.

### *Knockdown of KCNJ2 suppresses metastasis of OS cells *in vitro* and *in vivo

**Fig. 2:**
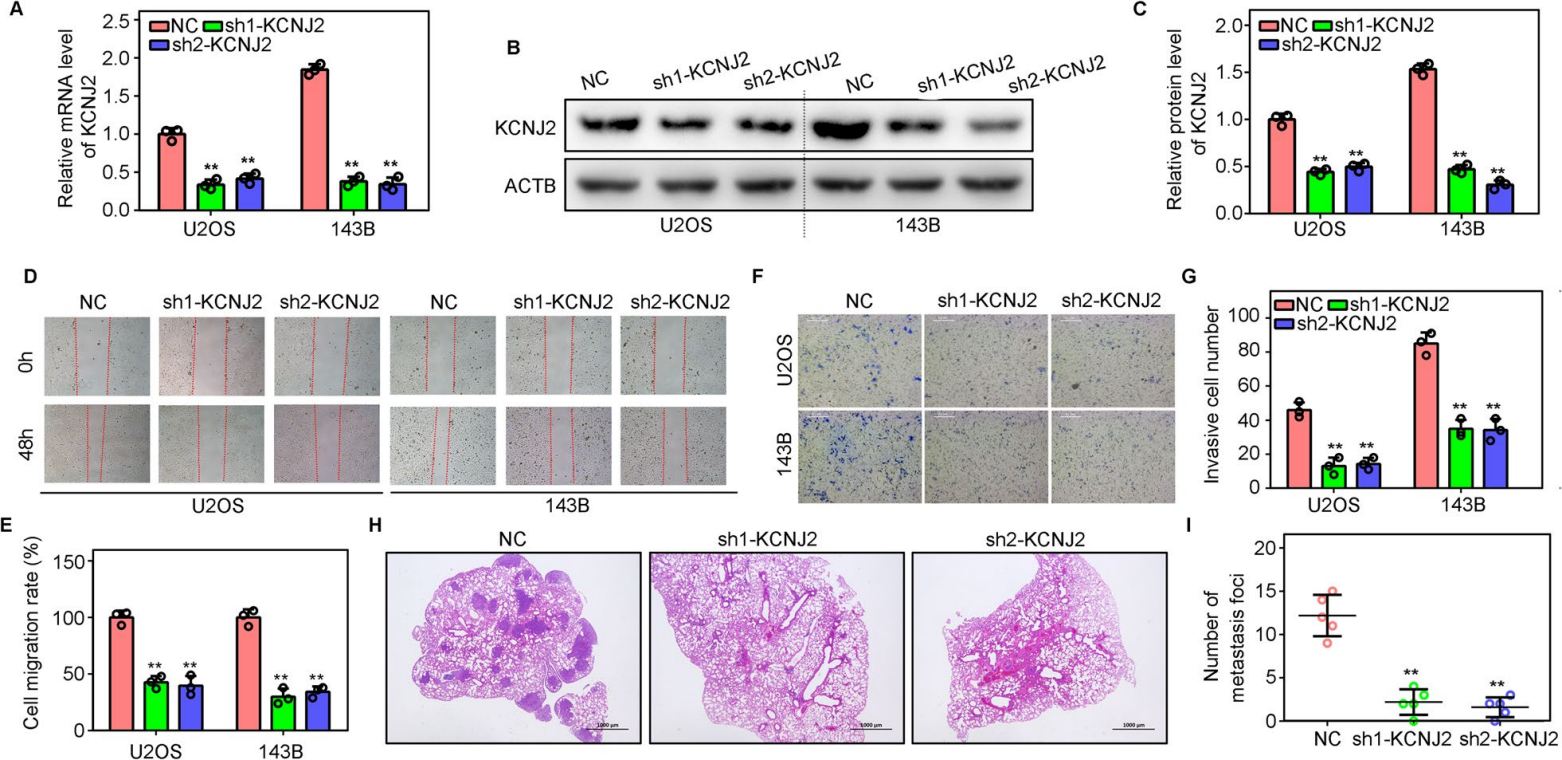
KCNJ2-inhibition represses the metastasis of OS cells. **A** Quantitative real-time polymerase chain reaction (qRT-PCR) was used to detect the mRNA levels of *KCNJ2* after transfection of sh1-KCNJ2 and sh2-KCNJ2. **B**, **C** Western blotting was used to detect the protein levels of KCNJ2 after transfection of sh1-KCNJ2 and sh2-KCNJ2, respectively. **D**, **E** Wound healing assays were used to detect the migration rate of OS cells after KCNJ2-inhibition. **F**, **G** Transwell assays were used to detect the invasion rate of OS cells after KCNJ2-inhibition. **H**, **I** Lung metastasis model was used to detect the metastasis of 143B cells in vivo after KCNJ2-inhibition. **, *P* < 0.01

We then determined the effect of KCNJ2 on OS cell metastasis. Two targeted *KCNJ2* shRNAs were used to suppress the expression of *KCNJ2* in U2OS and 143B cells (Fig. 2A–C). Results from the wound-healing assays indicated that U2OS and 143B cells with KCNJ2-inhibition exhibited lower migration rates than NC cells (Fig. 2D, E). Similarly, lower invasive ability was observed in U2OS and 143B cells with KCNJ2-inhibition (Fig. 2F–G). Moreover, we found that fewer metastatic foci were present in the lung tissues of mice injected with 143B cells with KCNJ2-inhibition (Fig. 2H, I). Taken together, these results indicated that the KCNJ2 knockdown suppressed OS cell metastasis.

### Overexpression of KCNJ2 promotes the metastasis of OS cells

**Fig. 3:**
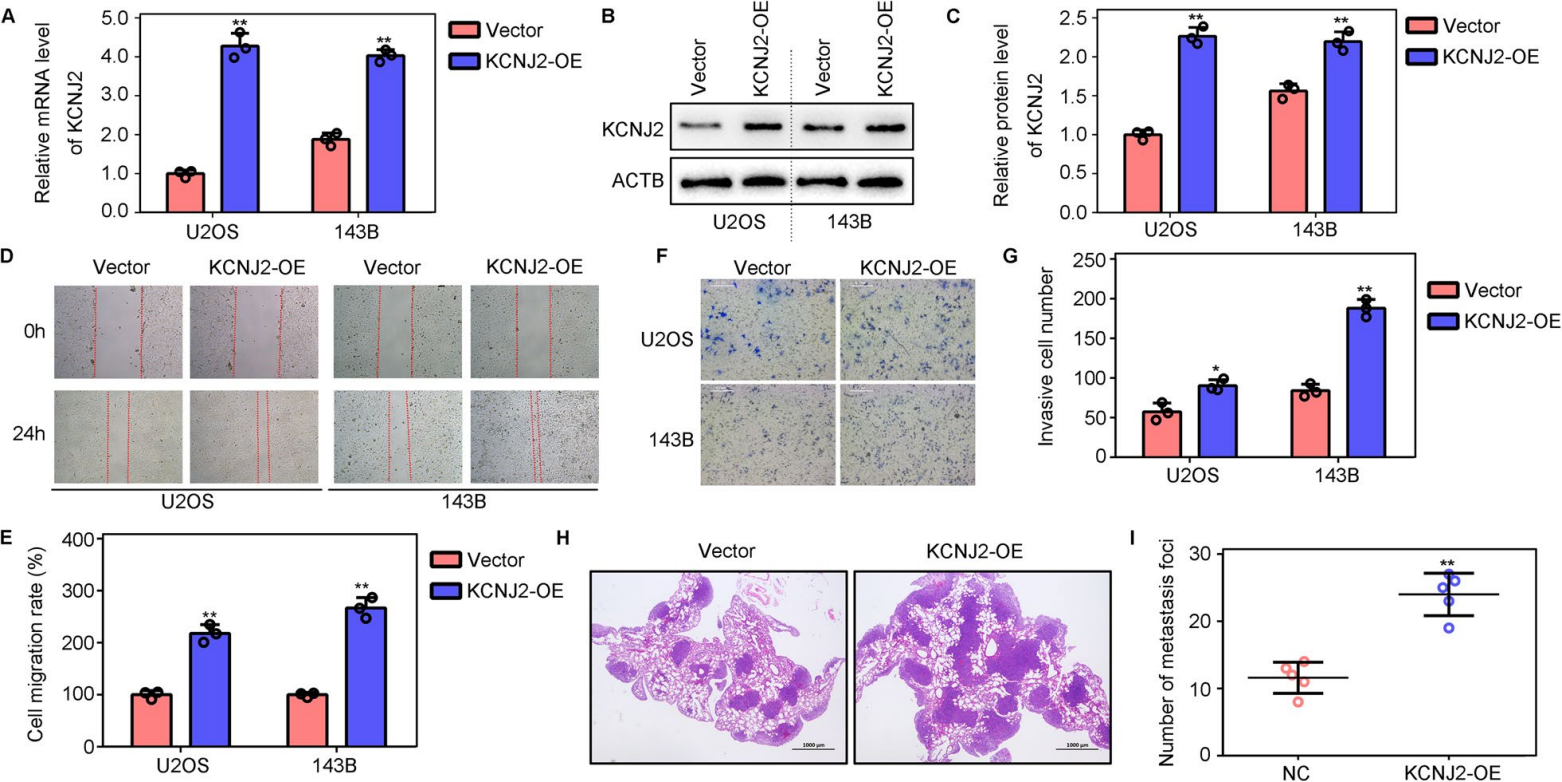
KCNJ2-overexpression increases the metastasis of OS cells. **A** Quantitative real-time polymerase chain reaction (qRT-PCR) was used to detect the mRNA levels of *KCNJ2* after *KCNJ2*-overexpression. **B**, **C** Western blotting was used to detect the protein levels of *KCNJ2* after *KCNJ2-*overexpression. **D**, **E** Wound healing assays were used to detect the migration rate of OS cells after *KCNJ2*-overexpression. **F**, **G** Transwell assays were used to detect the invasion rate of OS cells after *KCNJ2*-overexpression. **H**, **I** A lung metastasis model was used to detect the metastasis of 143B cells in vivo after *KCNJ2*-overexpression. **, *P* < 0.01

We constructed OS cells overexpressing KCNJ2 by transfection with a lentivirus (Fig. 3A–C) and used these cells to determine the effects of KCNJ2-overexpression on the metastasis of OS cells. W wound healing assay suggested that KCNJ2-elevation in U2OS and 143B cells promoted migration (Fig. 3D, E). Transwell assays indicated that KCNJ2-overexpressing U2OS and 143B cells exhibited stronger invasive ability than did NC cells (Fig. 3F–G). Furthermore, we found that KCNJ2-overexpressing 143B cells showed increased lung metastasis in vivo (Fig. 3H, I). This evidence indicated that the overexpression of KCNJ2 promotes metastasis of OS cells.

### KCNJ2 binds to HIF1α and is co-expressed with HIF1α in OS tissues

**Fig. 4:**
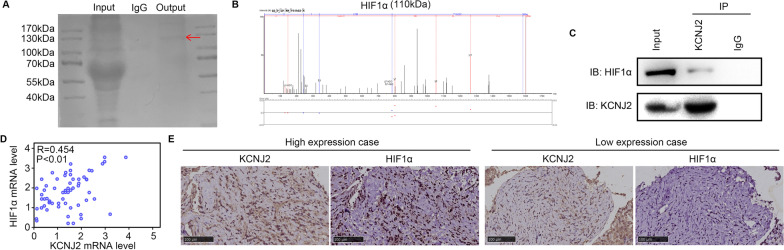
KCNJ2 binds to KCNJ2 and is co-expressed with KCNJ2 in OS tissues. **A** Coomassie brilliant blue staining exhibited the landscape of KCNJ2-interacting proteins. Input line was added the total proteins in 143B cells, IgG line was added the proteins enriched by IgG antibodies in 143B cells, while output line was added the proteins enriched by KCNJ2 antibodies in 143B cells. **B** Mass spectrometry identified HIF1α as KCNJ2 interacting protein of KCNJ2. For the figures of secondary mass spectrum, red lines mean water peak, while blue lines mean NH3 peak. **C** Immunoprecipitation demonstrated the interaction between KCNJ2 and HIF1α. **D** *KCNJ2* mRNA levels were co-expressed with *HIF1A* mRNA levels. **E** KCNJ2 protein levels were co-expressed with HIF1α protein levels

To analyze the molecular mechanisms by which KCNJ2 affects OS, we used an anti-KCNJ2 antibody to analyze proteins interacting with KCNJ2 in 143B cells. Mass spectrometry analysis indicated that HIF1α was highly abundant among the KCNJ2-interacting proteins (Fig. 4A, B). Similarly, immunoprecipitation results also indicated binding between KCNJ2 and HIF1α (Fig. 4C). Furthermore, we found that KCNJ2 was co-expressed with HIF1α at the mRNA (*R* = 0.454; Fig. 4D) and protein levels (Fig. 4E). Taken together, the results indicated that HIF1α binds to KCNJ2 and is co-expressed with KCNJ2 in OS tissues.

### KCNJ2-overexpression reduces the ubiquitination-mediated degradation of HIF1α

**Fig. 5:**
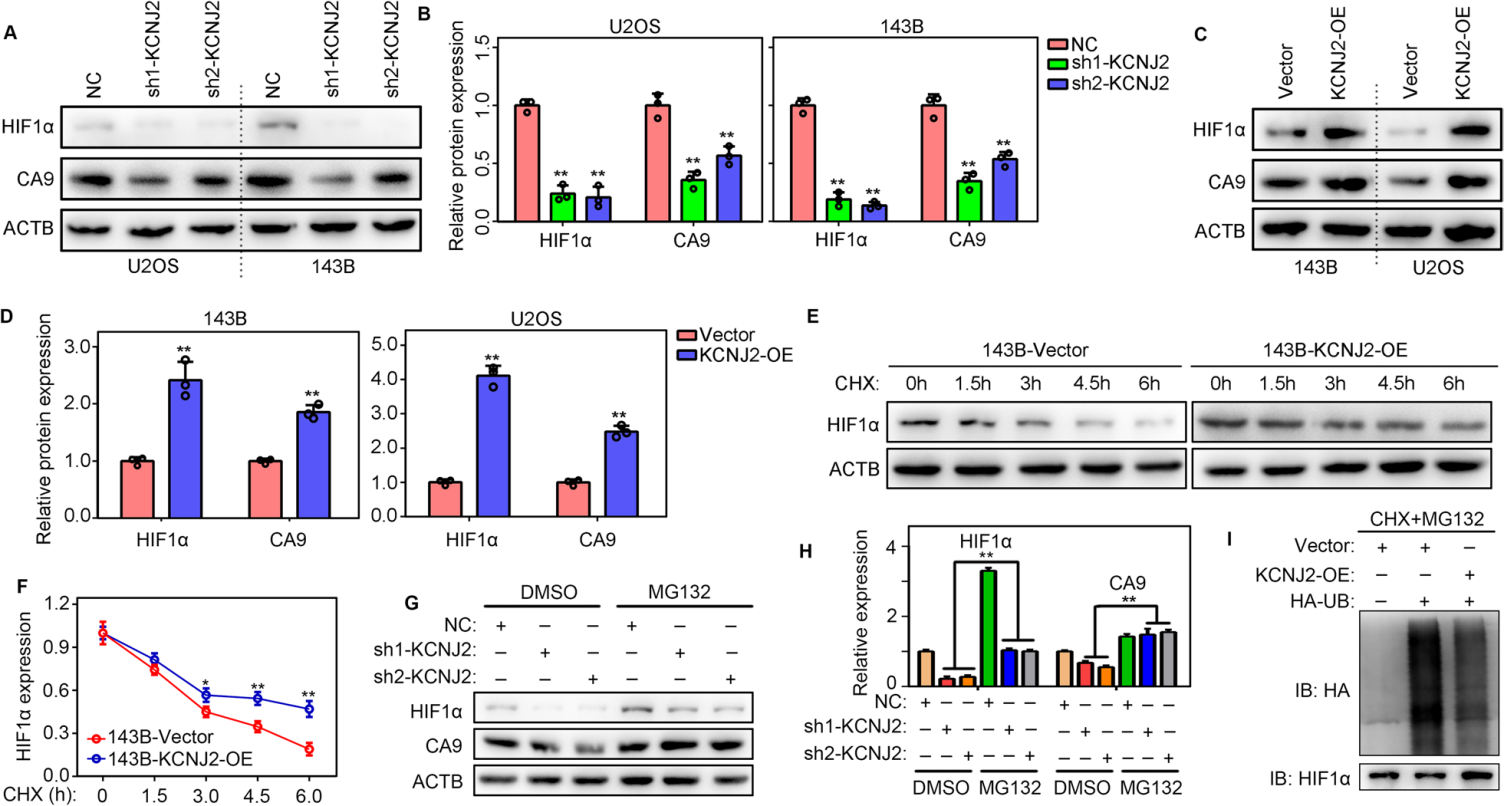
KCNJ2 reduced the ubiquitination degradation of HIF1α. **A**–**B** Expression of HIF1α and CA9 was detected by western blotting after KCNJ2-inhibition. **C**–**D** Expression of HIF1α and CA9 was detected by western blotting after KCNJ2-overexpression. **E**–**F** The degradation rate of HIF1α in 143B cells with empty vector and KCNJ2-overexpression. **G**–**H** Western blotting indicated that KCNJ2-inhibition markedly reduced HIF1α and CA9 expression in 143B cells, while the 26S protease inhibitor MG132 relieved this downregulation of HIF1α and CA9 that was induced by KCNJ2-inhibition. **I** Ubiquitination levels of HIF1α in 143B cells with empty vector and KCNJ2-overexpression. **, *P* < 0.01

We then determined whether KCNJ2 affected the expression of HIF1α. Western blotting demonstrated that protein levels of HIF1α, as well as its target, CA9, was reduced in U2OS and 143B cells (Fig. 5A, B). Similarly, KCNJ2 overexpression elevated HIF1α and CA9 expression in U2OS and 143B cells (Fig. 5C, D). Interestingly, the degradation rate of HIF1α was significantly reduced in 143B cells with KCNJ2-overexpression after CHX treatment (Fig. 5E, F). Moreover, we found that KCNJ2-inhibition markedly reduced HIF1α and CA9 expression in 143B cells, while the 26S protease inhibitor MG132 relieved this downregulation of HIF1α and CA9 that was induced by KCNJ2-inhibition (Fig. 5G, H). Furthermore, we found that KCNJ2 overexpression reduced ubiquitination-induced HIF1α degradation (Fig. 5I).

### Knockdown of HIF1α reduces the oncogenic effects of KCNJ2-overexpression on OS cell migration and invasion

**Fig. 6:**
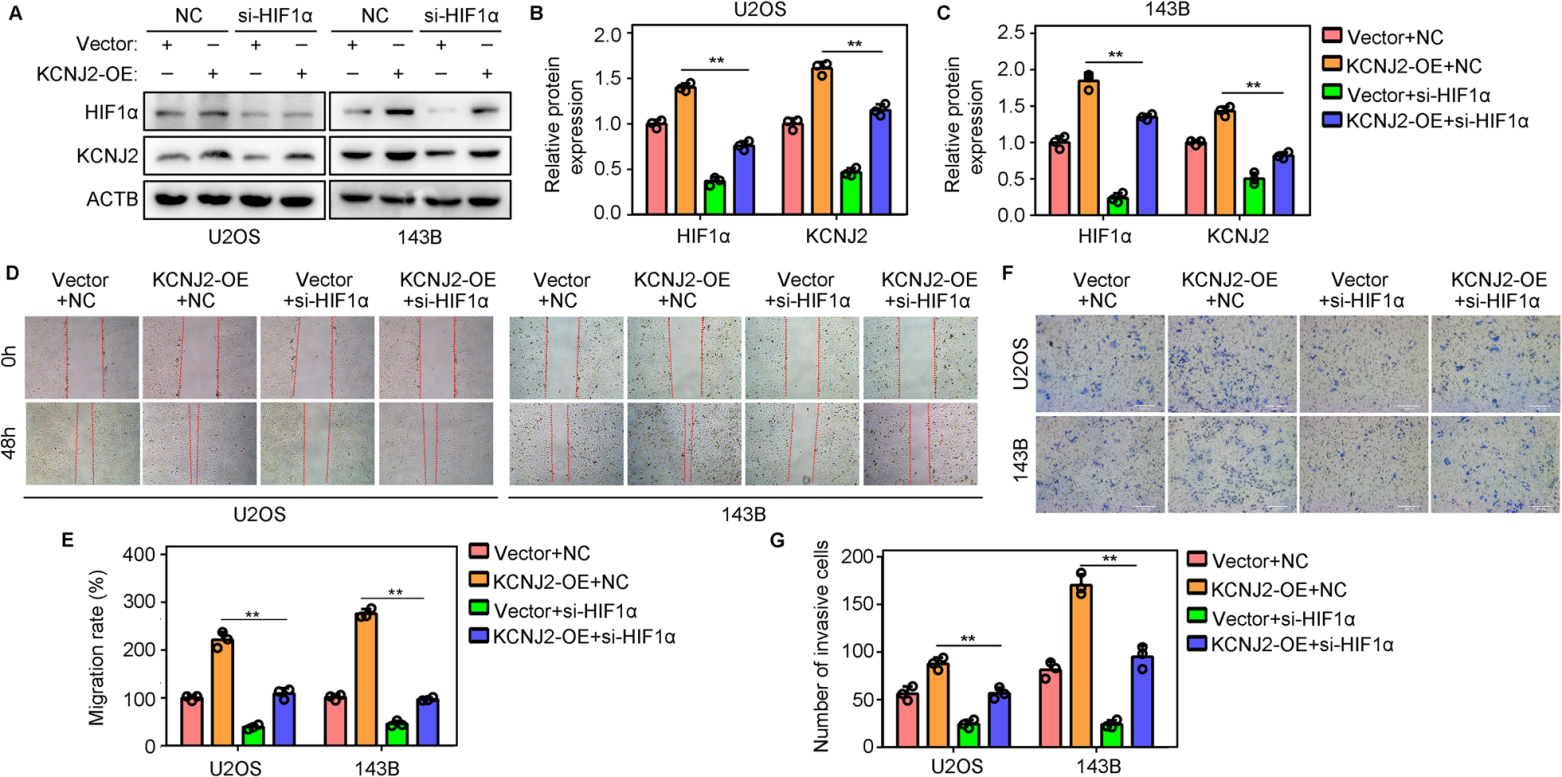
Knockdown of HIF1α reduces the oncogenic effects of KCNJ2-overexpression on OS cell. OS cells were treated as follows: negative control siRNAs (NC) + empty vector; NC + KCNJ-overexpression; targeting HIF1α siRNAs (si-HIF1α) + vector; si-HIF1α + KCNJ2-overexpression. **A**–**C** Protein levels of HIF1α and KCNJ2 were detected by western blotting in each group cells. **D**, **E** Wound healing assays were used to detect the migration rate of each group. **F**, **G** Transwell assays were used to detect the invasion rate of each group. **, *P* < 0.01

To determine whether HIF1α participates in KCNJ2-dependent progression, we reduced the expression of HIF1α in KCNJ2-overexpressing OS cells by transfecting the targeting HIF1α-siRNAs (Fig. 6A–C). The wound healing assay suggested that knockdown of HIF1α alleviated the pro-migration effects induced by KCNJ2-overexpression in U2OS and 143 B cells (Fig. 6D, E). Similarly, Transwell assays showed that the pro-invasion effects of KCNJ2-overexpression could be reversed by HIF1α-knockdown in U2OS and 143 B cells (Fig. 6F–G). This evidence indicated that knockdown of HIF1α reduced the oncogenic effects of KCNJ2-overexpression on OS cell migration and invasion.

### HIF1α regulates KCNJ2 transcription directly by binding to its promoter

**Fig. 7:**
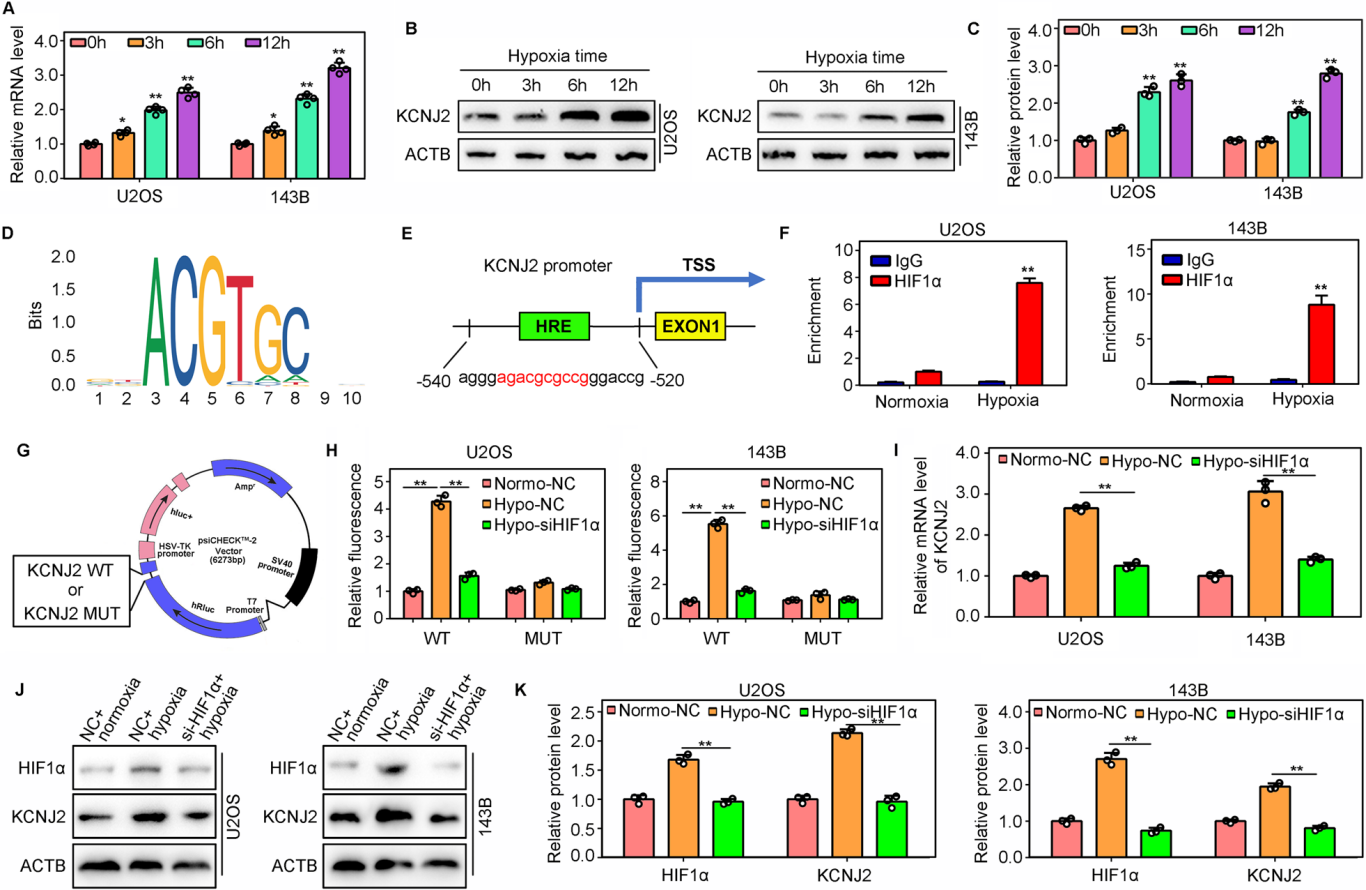
HIF1α directly regulates KCNJ2 transcription by binding to its promoter. **A** Quantitative real-time polymerase chain reaction (qRT-PCR) was used to detect the mRNA levels of *KCNJ2* in U2OS and 143B cells under hypoxic conditions. **B**, **C** Western blotting was used to detect the protein levels of KCNJ2 in U2OS and 143B cells under hypoxic conditions. **D** Motif of HIF1α. **E** Hypothetical binding site of HIF1α in the *KCNJ2* promoter (F) CHIP-qPCR was used to detect the binding site of HIF1α in the *KCNJ2* promoter. **G**, **H** Double luciferase reporter assays detected fluorescence intensity in NC and si-HIF1α cells transfected with wildtype/mutated plasmids under normoxia and hypoxia. **I** qRT-PCR was used to detect the mRNA levels of *KCNJ2* in NC and si-HIF1α cells under normoxic and hypoxic conditions. **J**, **K** western blotting was used to detect the protein levels of KCNJ2 in NC and si-HIF1α cells under normoxia and hypoxia. *, *P* < 0.05; **, *P* < 0.01

Interestingly, the mRNA (Fig. 7A) and protein levels (Fig. 7B, C) of KCNJ2 were markedly increased in U2OS and 143B cells after culturing under hypoxic conditions. Therefore, we investigated whether KCNJ2 was regulated by hypoxia-inducible factors. By matching the HIF1α motif (Fig. 7D) to the sequence of the KCNJ2 promoter, a binding site (− 526 to − 536) in the *KCNJ2* promoter was predicted to be bound by HIF1α (Fig. 7E). To verify this, a pair of specific primers was used to perform qRT-PCR for amplification of this sequence from the products of CHIP, using IgG and anti-HIF1α antibodies. The results indicated that the HIF1α binding sequence in the *KCNJ2* promoter was significantly amplified in the CHIP products obtained with an anti-HIF1α antibody, but not in those obtained with an IgG antibody (Fig. 7F). This phenomenon was more evident under hypoxic conditions (Fig. 7F). Moreover, we constructed fluorescein reporter plasmids with wildtype and mutated binding site sequences and transfected them into U2OS and 143B cells (Fig. 7G). Hypoxia increased the fluorescence intensity in U2OS and 143B cells transfected with the wildtype binding site sequence, while knockdown of HIF1α reduced the effects induced by hypoxia (Fig. 7H). However, these phenomena were not observed in U2OS and 143B cells transfected with the mutated binding site sequence (Fig. 7H). Finally, we found that hypoxia increased the mRNA (Fig. 7I) and protein levels (Fig. 7J, K) of KCNJ2 in U2OS and 143B cells, whereas knockdown of HIF1α reduced the effects induced by hypoxia. Thus, HIF1α directly regulates *KCNJ2* transcription by binding to its promoter.

## Discussion

OS is an aggressive malignancy in bone that grows rapidly and metastasizes in early stage [24]. Previous studies have indicated that hypoxia is a key OS characteristic, and induces OS cell metastasis and drug resistance [25]. HIF-1α is an important mediator of the tumor cell response to hypoxia, therefore, identification of HIF1α-regulating network would help the diagnosis and therapy of OS [26, 27]. We here indicated that KCNJ2 and HIF1α form a feedback loop and greatly promote OS cell metastasis.

KCNJ2 belongs to the classical subfamily of inward rectifying potassium channels. In neurons, skeletal muscle, cardiac myocytes, immune system cells, and carcinoma cells, KCNJ2 conducts an inward rectifying potassium current [28, 29]. Previous studies have indicated that KCNJ2 can promote the progression of a series of cancers, independent of the molecular mechanism of the inward rectifying potassium current. Liu et al*.* demonstrated that KCNJ2 expression was elevated in small-cell lung cancer tissues, and induced multiple drug resistance via Ras/MAPK pathways [19]. Chen et al*.* reported that KCNJ2 inhibition could repress the epithelial–mesenchymal transition of papillary thyroid carcinoma cells by elevating G protein subunit gamma 2 expression [30]. KCNJ2 can bind to and activate serine/threonine kinase 38, thereby promoting the invasion of gastric cancer cells [31]. In this study, we found that KCNJ2 levels were elevated in OS cells with high metastatic ability and in advanced OS tissues. A shorter overall survival rate was observed in patients with OS who expressed high levels of KCNJ2. Knockdown of KCNJ2 repressed the metastasis of OS cells, whereas KCNJ2-elevation induced the opposite effects. This provides evidence that KCNJ2 acts as an oncogene in OS progression.

To understand the molecular mechanism underlying the effects of KCNJ2, we performed immunoprecipitation and found that KCNJ2 bound to HIF1α. Furthermore, we found that KCNJ2 is co-expressed with HIF1α in OS tissues. Knockdown of KCNJ2 decreased HIF1α expression and its downstream protein, CA9, while overexpression of KCNJ2 elevated HIF1α and CA9 expression. Previous studies indicated that most HIF-1α protein would be degraded by the von Hippel–Lindau tumor suppressor under normoxic conditions, while a few HIF-1α protein would escape from the degradation with the help of a series of protective proteins [32]. Given the interaction observed between KCNJ2 and HIF1α, we considered whether KCNJ2 was a protective protein of HIF1α. Interestingly, KCNJ2 knockdown increased the degradation rate of HIF1α. As HIF1α is strongly degraded via ubiquitination, we used MG132, a 26S proteinase inhibitor, to treat OS cells. The results indicated that MG132 increased the expression of HIF1α in KCNJ2-knockdown cells. Furthermore, reduced ubiquitination was observed in the KCNJ2-overexpressing 143B cells. Thus, this study revealed that KCNJ2 may protect HIF1α by helping it to escape ubiquitination.

Interestingly, we found that under hypoxic conditions, *KCNJ2* mRNA and protein levels were increased. Previous studies have indicated that target genes can be transcribed more efficiently when HIFs bind to their promoter regions. To date, a series of HIF target genes have been identified in OS, such as the genes encoding GATA-binding protein 1 [33] and SUMO-specific peptidase 1 [34]. These HIF target genes are involved in metastasis and drug resistance in OS. Therefore, we considered whether a member of the HIF family could regulate *KCNJ2*. Consistent with our hypothesis, a binding site for HIF1α was found in the *KCNJ2* promoter, and we showed that HIF1α binds directly to the *KCNJ2* promoter, enhancing transcription of this gene. This evidence indicated that KCNJ2/HIF1α forms a feedback loop that markedly amplifies the downstream signal of HIF1α.

In conclusion, our study demonstrated that KCNJ2 and HIF1α form a positive feedback loop that promotes OS metastasis. This suggests that KCNJ2 may be a promising biomarker and treatment target for OS. Blocking hypoxic signaling via inducing HIF1α degradation in OS cells may be achieved through inhibition of KCNJ2. In the future, we will proceed to develop inhibitors for KCNJ2 for OS. Actually, even overexpressed in OS cell lines, KCNJ2 is expressed in many cell types and organs. Therefore, we considered, combining with a biological delivery system that targets hypoxic areas of the tumor, the KCNJ2 inhibitors would exhibit more effective for anti-OS and evade nonspecific toxicity to other tissues.

## Data Availability

The datasets generated and/or analyzed during the current study are available from the corresponding authors upon reasonable request.
